# Association between *TLR9* rs5743836 polymorphism and risk of recurrent venous thromboembolism

**DOI:** 10.1007/s11239-017-1491-3

**Published:** 2017-03-20

**Authors:** Abrar Ahmad, Kristina Sundquist, Bengt Zöller, Peter J. Svensson, Jan Sundquist, Ashfaque A. Memon

**Affiliations:** 1 Department of Clinical Sciences, Center for Primary Health Care Research, Lund University/Skåne University Hospital, Malmö, Sweden; 2 Department of Coagulation Disorders, Skåne University Hospital, Lund University, Malmö, Sweden

**Keywords:** Recurrent VTE, Multivariate analysis, Toll-like receptor

## Abstract

Recent gene knockout studies on mice have shown the role of toll-like receptor 9 (*TLR9*) in resolution of venous thromboembolism (VTE) through sterile inflammation. However, the role of a putative functional *TLR*9 polymorphism (rs5743836) in risk assessment of VTE recurrence remains unknown. The aim of our study was to investigate the *TLR9* rs5743836 polymorphism in VTE patients and its association with the risk of VTE recurrence. We analyzed *TLR9* rs5743836 polymorphism in Malmö thrombophilia study patients; a prospective follow-up study of 1465 VTE patients by Taqman PCR. From a total of 1465 VTE patients, those who had VTE before inclusion and those who died or had VTE recurrence during anticoagulant treatment were excluded (n = 415). Cox regression analyses were performed on the remaining 1050 VTE patients, including 126 (12.5%) patients that had recurrent VTE during follow-up period. *TLR9* polymorphism was significantly associated with higher risk of VTE recurrence in female patients (HR 3.46, 95% CI 1.06–11.33) independent of acquired risk factors for VTE, family history, risk of thrombophilia and deep vein thrombosis (DVT) location. Similarly, in unprovoked VTE patients, *TLR*9 polymorphism was significantly associated with higher risk of VTE recurrence in female patients (HR 5.94, 95% CI 1.25–28.13) after adjusting for family history, risk of thrombophilia and DVT location. No association between *TLR9* polymorphism and risk of VTE recurrence was found in male patients. Our results suggest that *TLR9* rs5743836 polymorphism is an independent risk factor for VTE recurrence in female patients but not in males.

**Electronic supplementary material:**

The online version of this article (doi:10.1007/s11239-017-1491-3) contains supplementary material, which is available to authorized users.

## Introduction

Venous thromboembolism (VTE) consists of two related conditions: deep vein thrombosis (DVT) and pulmonary embolism (PE) [1]. Primarily, it was considered to be a complication of hospitalization after major surgery. However, prior epidemiological studies have reported that about half of the patients, which had been diagnosed with VTE, were those who had never been hospitalized nor had any major illness [1, 2]. VTE is a potentially lethal disease that occurs with an incidence rate of 1–2 cases per 1000 person-years [2, 3]. About two-thirds of the patients diagnosed with symptomatic VTE had DVT and one-third had PE [2]. The mortality rate after 30 days of diagnosis with DVT was reported as 4.6%, with PE as 9.7 and 6.4% for patients diagnosed with VTE (both DVT and PE) [4]. VTE is a chronic disease and patients that have experienced one episode of VTE are always at the risk of recurrence and the risk is highest during the first 6–12 months [5]. The cumulative rate of VTE recurrence increases with time; 17.5% after 2 years, 24.96% after 5 years and rises up to 30.3% after 8 years from the first diagnosis with primary VTE [6]. Patients with unprovoked VTE (without known acquired risk factors for VTE, e.g., older age, immobilization, trauma, major surgery, female hormone therapy, pregnancy etc.) are at higher risk of VTE recurrence as compared to provoked VTE (patients with acquired risk factors) [7].

VTE patients are treated with standard treatment protocol, i.e., anticoagulant therapy for several months which protects patients from VTE recurrence, however, at the cost of severe bleeding [8, 9]. Regardless of the several identified risk factors, such as sex, D-dimers level and residual thrombosis, it is still difficult to precisely predict the probability of VTE recurrence after stopping the anticoagulation treatment [8–10]. Moreover, anticoagulants prevent the formation of new blood clots but do not remove the existing thrombi, which undergo a slow process of natural resolution by organization and vein recanalization [11].

Although a number of genes are identified as risk factors for VTE, the major portion of the heritability remains unknown. Twins and familial studies have reported that the heritability cause of VTE is up to 60%, and most of these genetic factors are still unknown [12, 13]. It is therefore clinically relevant to identify new biomarkers that allow early prediction of patients at risk of VTE recurrence in order to tailor anti-coagulant therapy.

VTE pathophysiology is now better understood following several experimental studies. It is believed that inflammation plays a major role in VTE pathophysiology [14]. Studies conducted in the 1970s using radiolabel leukocytes showed that there is an uptake of white blood cells in the venous thrombus [15, 16]. Furthermore, plasma levels of C-reactive protein (CRP), a prominent acute-phase reactant and inflammatory marker, are known to increase in DVT patients [17]. Moreover, clinically it is apparent that VTE patients manifest the cardinal signs of inflammation (heat, redness, pain and swelling). Thus, inflammation is considered to have a pivotal role in VTE formation.

Venous thrombus formation, propagation and its dissolution is a balance between two mechanisms, i.e. coagulation and innate immune mechanisms [14]. DVT resolution is an inflammatory process (known as sterile inflammation) which is similar to that of sterile wound healing. It occurs naturally through a process of tissue organization including neovascularization [14, 18]. Fibrinogen, in venous thrombus, along with its degradation products is present in abundance and these molecules stimulate the activation of the innate immune system to produce cytokines and chemokines that are involved in the sterile inflammatory process [19].

TLRs are important members of the innate immune system. Among the TLR family, *TLR9* has been studied for its role in VTE resolution. Leukocytes (monocytes, neutrophils, lymphocytes and dendritic cells) express *TLR9* on their membranes [20, 21]. Animal studies show that deletion of the *TLR9* gene severely restricts the VTE resolution process, which suggests that *TLR9* is an important player in venous thrombus resolution by modulating sterile inflammation [22, 23]. *TLR9* gene has been found to be polymorphic in several diseases [24–27]. One of the putative functional polymorphism is *TLR9* rs5743836 polymorphism (−1237T/C polymorphism), which is located within the promoter region of *TLR9* gene and has been studied in several diseases including cardiovascular, cancers and autoimmune diseases [27–30]. Since this polymorphism (*TLR*9 rs5743836 polymorphism) is shown to affect the transcription of *TLR9* gene, and *TLR9* gene is involved in VTE resolution, it is worthwhile to investigate the role of *TLR9* rs5743836 polymorphism in recurrent VTE patients and its association with the risk of VTE recurrence. To our knowledge, this is the first study in which *TLR9* rs5743836 polymorphism has been studied in a well-established VTE cohort to investigate its association with the risk of VTE recurrence. Novel knowledge will therefore be obtained with the potential to influence future risk assessments and pharmacological preventive measures.

## Materials and methods

### Study subjects

A prospective population-based study of 1465 consecutive unselected VTE patients [Malmö thrombophilia study (MATS)] was performed at Skåne University Hospital. MATS is a well characterized cohort including VTE patients that were followed after inclusion (March 1998), until VTE recurrence or death of the patient or end of the study (December 2008) [31, 32]. For all MATS patients, VTE events prior to inclusion, location of DVT, immobilization and cast therapy, hospitalization, surgical intervention, malignancies that were diagnosed previously or at diagnosis of VTE, use of contraceptive pills, hormonal therapy, pregnancy and postpartum period (first 6 weeks after delivery), family history of VTE (history of VTE in first-degree relatives), and VTE recurrence during the follow-up period were recorded. All hospital records for VTE patients were screened by a research nurse. The rate of consensual participation in this study was 70%. The remaining patients (30%) were excluded from the study because they refused to participate or did not give consent for the blood sampling or could not complete the questionnaire for the risk factor analysis due to dementia, language problems or presence of other severe diseases.

The inclusion criteria in MATS were: diagnosis of DVT and/or PE. VTE diagnosis was objectively confirmed by phlebography, computed tomography (CT), duplex ultrasonography, lung scintigraphy or magnetic resonance imaging (MRI).

All MATS patients were treated according to the standard treatment protocol of Malmö University Hospital, i.e., all patients were initially treated with low molecular weight heparin or unfractionated heparin and then with warfarin as an oral anticoagulant. The hospital treatment protocol recommended therapy for 3–6 months for first-time VTE, with consideration of extended treatment in case of recurrent VTE. A total of 18% of patients were treated for >1 year or for life. Thrombophilia was defined as presence of the factor V Leiden (FVL) mutation (rs6025) or factor II G20210A mutation (rs1799963), or a level below the laboratory reference range of protein C [<0.7 kilo international unit (kIU/L)], free protein S (women < 0.5 kIU/L, men < 0.65 kIU/L) or antithrombin (<0.82 kIU/L) in patients without warfarin treatment.

Follow-up period (Mean ± SD, 3.9 ± 2.5) was counted in years after stopping the anticoagulant treatment until the diagnosis of VTE, death of the patient or the end of study (December 2008). This study was approved by the ethical committee of Lund University. All the participants provided written permission before their inclusion in the study according to the declaration of Helsinki.

### Laboratory methods

DNA from the whole blood was extracted by using QiAmp 96 DNA Blood Kit (Qiagen, Hilden, Germany) according to the manufacturer’s instructions. Genotyping of *TLR9* rs5743836 polymorphism was performed by TaqMan® SNP Genotyping Assay according to the manufacturer’s protocol (Applied Biosystems, Life Technologies Corporation, Carlsbad, CA, USA). To summarize, a polymerase chain reaction (PCR) master mix was prepared as 2.5 µL Taqman master mix, 0.25 µL Taqman gene-specific assay (VIC and FAM probes for *TLR9* rs5743836 polymorphism) and 0.25 µL deionized water. Master Mix (3 µL) was added to each well in 384 wells PCR plate followed by addition of 10 ng DNA. PCR plate was vortexed and centrifuged at 1000 rpm (revolutions per minute) for 30 s. BioRad CFX384 real-time PCR (1000 Alfred Nobel Drive Hercules, California 94547 USA), according to manufacturer’s instructions, was used for polymorphism analysis with the following temperature conditions: 95 °C for 10 min followed by 40 × (92 °C for 15 s, 60 °C for 1 min). Different alleles of *TLR9* rs5743836 polymorphism were determined by using BioRad CFX manager software.

### Analysis of known thrombophilic variants

FVL and factor II G20210A mutations in DNA were analyzed by TaqMan allele discrimination assays (Applied Biosystems) as described previously [33]. Analysis of free Protein S was performed by latex immunoassay with Coamatic^®^ Protein S-Free (Chromogenix, Haemochrom Diagnostica AB, Gothenburg, Sweden) [34]. Protein C levels were analyzed by chromogenic method using the Berichrom^®^ Protein C reagent (Siemens Healthcare Diagnostics, Upplands Väsby, Sweden) [35]. For antithrombin analysis, thrombin-based method using Berichrom Antithrombin (Siemens Healthcare Diagnostics) was used [36]. BCS-XP coagulation analyzer (Siemens Healthcare Diagnostics) was used to perform these analyses.

### Statistical analysis

Statistical analyses were performed by using SPSS version 21 (IBM, Armonk, NY, USA). Dichotomous variables were compared by Chi square test or Fisher’s exact test, where appropriate and continuous variables were compared by Mann–Whitney *U* test. Interaction term analysis was performed to test for an interaction between *TLR*9 rs5743836 polymorphism and gender of the patients. Kaplan–Meier survival curves for time to recurrent VTE by *TLR9* rs5743836 polymorphism genotypes were plotted and the log-rank test was used to compare recurrence-free survival between genotypes. Hardy–Weinberg equilibrium analysis was performed to observe the genotypic distribution. Univariate and multivariate Cox regression analyses were performed using Cox proportional hazards models, after adjusting for location of DVT, family history of VTE, mild and severe thrombophilia and acquired risk factors for VTE. For each group of patients, hazard ratios (HRs) with 95% confidence intervals (CIs) were calculated. Multivariate Cox regression analyses were performed as sensitivity analyses by including all VTE patients, with exception for those which had thrombotic events before inclusion. The follow-up period for sensitivity analyses was calculated from the time of inclusion and was adjusted for the duration of anticoagulation treatment.

## Results

### Clinical data of the patients

From a total of 1465 patients, those who had thrombotic events before inclusion (n = 154) were excluded. For the remaining patients (n = 1311), 148 (11.3%) had VTE recurrence during the follow-up period. Frequency of FVL mutation was higher in patients with recurrent VTE (40.8%) as compared to those with non-recurrent VTE (28.5%) (P = 0.002). Of the patients with recurrent VTE, 32.4% had a family history of VTE as compared to 23.5% in non-recurrent VTE (P = 0.024). Whereas, no significant difference was observed among recurrent and non-recurrent VTE patients in age, sex, deep vein thrombosis (DVT), pulmonary embolism (PE), body mass index (BMI), protein C, protein S and antithrombin deficiency (P > 0.05) Table 1.

**Table 1:** Characteristics of studied population including the distribution of *TLR9* rs5743836 polymorphism genotypes stratified by recurrent and non-recurrent status

Parameters	Mean (±SD) or n(%)		Total n (%)	P-value^¶^
	Non recurrent VTE n (%)	Recurrent VTE n (%)		
Age at inclusion				
Years (mean ± SD)	62.9 ± 17.5	61.3 ± 15.3	62.7 ± 17.3	0.087*
Gender				
Male	565 (48.6)	78 (52.7)	643 (49.0)	0.383
Female	598 (51.4)	70 (47.3)	668 (51.0)	
BMI				
Mean ± SD	26.6 ± 4.7	27.4 ± 5.1	26.6 ± 4.8	0.110*
PE				
PE	343 (29.5)	45 (30.4)	388 (29.6)	0.848
No PE	820 (70.5)	103 (69.6)	923 (70.4)	
DVT + PE				
DVT	820 (70.5)	103 (69.6)	923 (70.4)	0.306
PE	277 (23.5)	32 (21.6)	309 (23.6)	
DVT + PE	66 (5.7)	13 (8.8)	79 (6)	
Malignancy				
Yes	140 (12.1)	13 (8.8)	153 (11.7)	0.278
No	1020 (87.9)	135 (91.2)	1155 (88.3)	
Protein C deficiency				
Yes	16 (1.6)	0 (0.0)	16 (1.4)	0.242
No	1009 (98.4)	1136 (100.0)	1145 (98.6)	
Protein S deficiency				
Yes	20 (2.0)	1 (0.7)	21 (1.8)	0.499
No	998 (98.0)	135 (99.3)	1133 (98.2)	
Factor V mutations				
Yes	330 (28.5)	60 (40.8)	390 (29.9)	**0.002**
No	829 (71.5)	87 (59.2)	916 (69.9)	
Factor II mutations				
Yes	39 (3.9)	9 (7.0)	48 (4.2)	0.104
No	969 (96.1)	120 (93.0)	1089 (95.8)	
Antithrombin deficiency				
Yes	12 (1.2)	1 (0.7)	13 (1.1)	0.726
No	1013 (98.8)	135 (99.3)	1148 (98.9)	
Family history				
Yes	269 (23.5)	47 (32.4)	316 (24.5)	**0.024**
No	875 (76.5)	98 (67.6)	973 (75.5)	
TLR9 (rs5743836)				
TT	853 (74.0)	112 (76.2)	965 (74.3)	0.799
TC	271 (23.5)	31 (21.1)	302 (23.2)	
CC	28 (2.4)	4 (2.7)	32 (2.5)	
TT and TC	1124 (97.6)	143 (97.3)	1267 (97.5)	0.777

DNA was not enough for genotyping in 12 samples for *TLR9* rs5743836 polymorphism. P-value, Chi square test until unless indicated

significant P-values are shown in bold letters

*DVT* deep vein thrombosis, *PE* pulmonary embolism, *BMI* body mass index

*Mann–Whitney *U* test

^¶^comparing non-recurrent with recurrent VTE

*TLR9* rs5743836 polymorphism has three genotypic forms, homozygous wild type (TT), heterozygous (TC) and homozygous mutated form (CC). In data analysis, all three genotypic forms were analyzed separately as well as by combining homozygous wild type and heterozygous form (Table 1).

In Hardy–Weinberg equilibrium analyses, genotypic distributions in *TLR*9 rs5743836 polymorphism did not deviate significantly (P > 0.05).

### *TLR9* rs5743836 polymorphism and risk of VTE recurrence

For the recurrence analyses of the patients (n = 1311), those who had recurrence or died during anticoagulant treatment were excluded (n = 261). Analyses were performed on the remaining 1050 patients including 126 (12%) recurrent VTE patients.

For *TLR9* rs5743836 polymorphism, univariate and multivariate Cox regression analyses were performed with individual genotypes (TT, TC and CC) to investigate their association with the risk of VTE recurrence. In the whole population, there was no significant association between *TLR*9 rs5743836 polymorphism and risk of VTE recurrence (HR 1.12, CI 0.41–3.04, P = 0.827 and HR 0.99, CI 0.36–2.70, P = 0.981) on Uni- and multi-variate Cox regression analyses respectively. However, inclusion of an interaction term between *TLR9* rs5743836 polymorphism and gender of the patients in the model showed a modifying effect of gender on *TLR*9 polymorphism (*TLR*9 polymorphism × gender: HR 2.49, 95% CI 1.08–5.74, P = 0.032). After stratification of data, according to gender, a significant association between *TLR9* rs5743836 polymorphism and risk of VTE recurrence was found in female patients on univariate Cox regression analysis (HR 3.60 and 95% CI 1.11–11.61, P = 0.033) and on multivariate Cox regression analysis after adjusting for location of DVT, family history of VTE, mild and severe thrombophilia and acquired risk factors for VTE (HR 3.46, CI 1.06–11.33, P = 0.040). Similar results were found when we combined the T allele containing genotypes (TT and TC) and compared with homozygous mutated genotype (CC); only female patients having *TLR9* rs5743836 polymorphism were at higher risk of VTE recurrence (HR 3.44, CI 1.07–11.0, P = 0.037 and HR 3.36, CI 1.04–10.88, P = 0.043 on univariate and multivariate Cox regression analyses respectively) (Table 2).

**Table 2:** Uni- and multivariate analyses of *TLR9* rs5743836 polymorphism in recurrent VTE patients

Genotypes	All patients				Men				Women			
	Univariate HR (95% CI)	P	Multivariate HR (95% CI)	P*	Univariate HR (95% CI)	P	Multivariate HR (95% CI)	P*	Univariate HR (95% CI)	P	Multivariate HR (95% CI)	P*
rs5743836												
TT	Reference		Reference		Reference		Reference		Reference		Reference	
TC	0.84 (0.54–1.30)	0.431	0.81 (0.52–1.26)	0.348	0.58 (0.30–1.10)	0.096	0.58 (0.30–1.12)	0.104	1.20 (0.67–2.17)	0.537	1.13 (0.62–2.08)	0.69
CC	1.12 (0.41–3.04)	0.827	0.99 (0.36–2.70)	0.981	0.34 (0.05–2.46)	0.286	0.34 (0.05–2.45)	0.282	3.60 (1.11–11.61)	**0.033**	3.46 (1.06–11.33)	**0.040**
TT&TC	Reference		Reference		Reference		Reference		Reference		Reference	
CC	1.16 (0.43–3.15)	0.766	1.04 (0.38–2.83)	0.941	0.38 (0.05–2.77)	0.342	0.38 (0.05–2.78)	0.342	3.44 (1.07–11.0)	**0.037**	3.36 (1.04–10.88)	**0.043**

significant P-values are shown in bold letters

*P  adjusted for acquired risk factors of VTE, family history of VTE, mild and severe thrombophilia and location of VTE

**Fig. 1:**
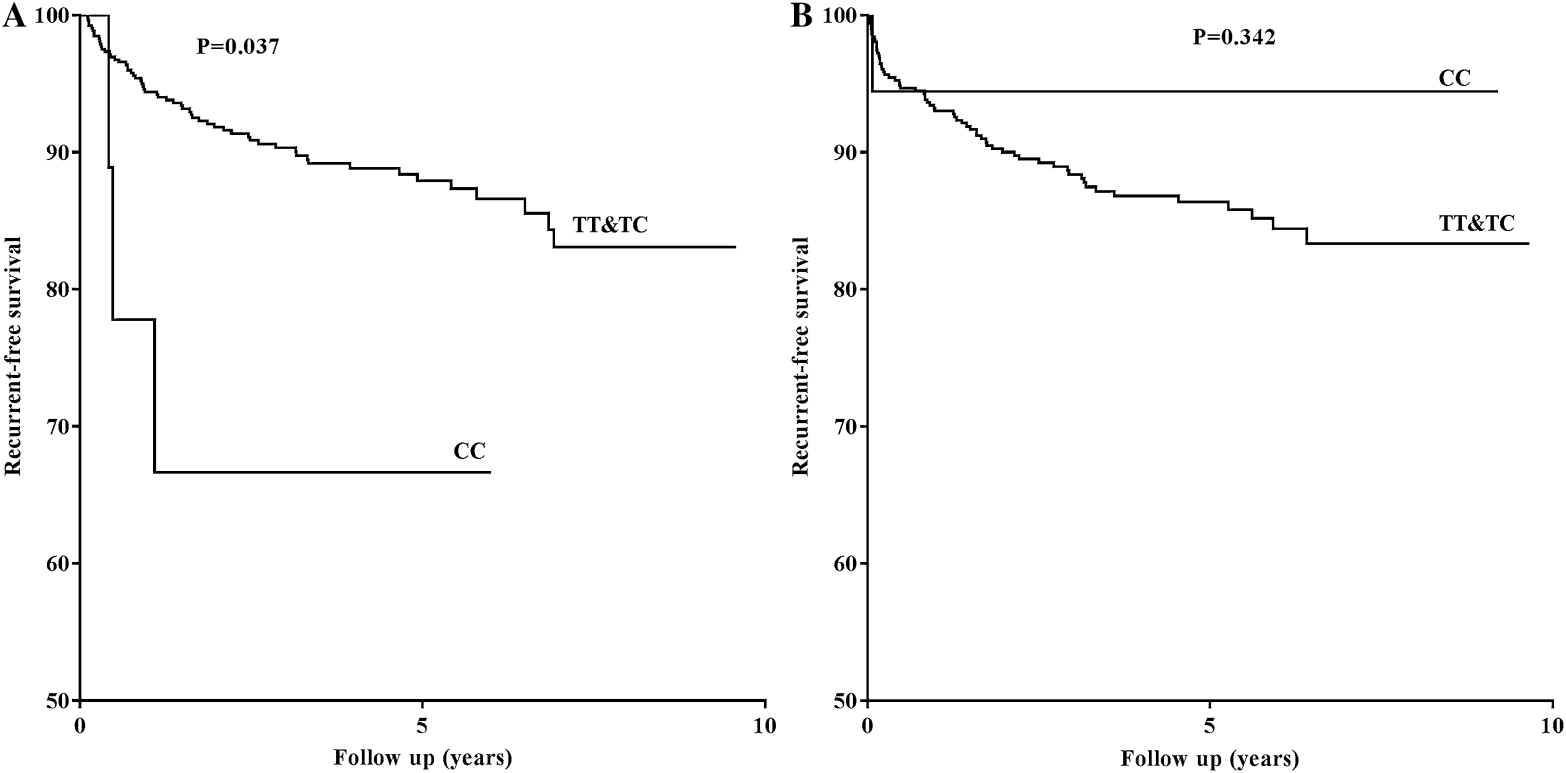
Survival curves representing the different genotypes in *TLR9* rs5743836 polymorphism and their association with risk of VTE recurrence in female (**a**) and male patients (**b**). P = log-rank test

A survival analysis by Kaplan–Meier curve was performed to analyze whether *TLR*9 rs5743836 polymorphism influences recurrence-free survival. Patients having T and C alleles were compared and results showed a significant difference in recurrence-free survival (Fig. 1a, Log-rank test, P = 0.037) in female patients. Female patients having CC genotype had significantly shorter recurrence-free survival as compared to TT and TC genotypes, whereas no significant difference was observed between different genotypes and risk of VTE recurrence in male patients (Fig. 1b, Log-rank test, P = 0.342).

### *TLR9* rs5743836 polymorphism and risk of VTE recurrence in patients with unprovoked first VTE

We also performed a sub-analysis on unprovoked first VTE patients (n = 618); patients with a recorded acquired risk factor for VTE, i.e., immobilization or cast therapy within the last month, use of contraceptives pills, surgical intervention, malignancies diagnosed prior to or at diagnosis of the first VTE event, current pregnancy and postpartum period (first 6 weeks after delivery) and female hormone therapy were excluded from this analysis. Our results show an association between *TLR*9 rs5743836 polymorphism and risk of VTE recurrence, only in female unprovoked VTE patients, though this association did not reach statistical significance on univariate Cox regression analysis (HR 3.79, CI 0.89–16.04, P = 0.071). However, on multivariate Cox regression analysis, after adjusting with location of DVT, family history of VTE, mild and severe thrombophilia, *TLR*9 polymorphism was significantly associated with the risk of VTE recurrence (HR 5.94, CI 1.25–28.13, P = 0.025) (Table 3).

**Table 3:** Uni- and multivariate analyses of *TLR9* rs5743836 polymorphism in unprovoked recurrent VTE patients

Genotypes	All unprovoked VTE patients				Unprovoked women			
	Univariate HR (95% CI)	P	Multivariate HR (95% CI)	P*	Univariate HR (95% CI)	P	Multivariate HR (95% CI)	P*
rs5743836								
TT	Reference		Reference		Reference		Reference	
TC	0.91 (0.54–1.53)	0.723	0.92 (0.54–1.56)	0.746	1.27 (0.61–2.63)	0.519	1.13 (0.53–2.42)	0.756
CC	0.65 (0.16–2.67)	0.553	0.60 (0.14–2.46)	0.474	3.79 (0.89–16.04)	0.071	5.94 (1.25–28.13)	**0.025**
TT&TC	Reference		Reference		Reference		Reference	
CC	0.67 (0.16–2.72)	0.573	0.61 (0.15–2.50)	0.491	3.57 (0.86–14.94)	0.081	5.79 (1.23–27.19)	**0.026**

significant P-values are shown in bold letters

*P adjusted for family history of VTE, mild and severe thrombophilia and location of VTE

Furthermore, sensitivity analyses were performed including all MATS patients (n = 1311) except those that had VTE before inclusion (n = 154). The follow-up time for these analyses was calculated from the time of inclusion and was adjusted for duration of anticoagulant treatment. Multivariate Cox regression analyses (adjusted for duration of anticoagulant treatment, mild and severe thrombophilia, location of DVT, family history and acquired risk factors for VTE), showed that female patients with *TLR9* rs5743836 polymorphism have significantly higher risk of VTE recurrence as compared to patients with wild-type genotype (HR 3.44, CI 1.05–11.26, P = 0.042) (Table 1 in the Supplementary Appendix).

## Discussion

In the current study, we have analyzed *TLR9* rs5743836 polymorphism for its role in the risk assessment of VTE recurrence. Our results show that *TLR9* rs5743836 polymorphism is significantly associated with higher risk of VTE recurrence in female patients, independent of known clinical risk factors associated with VTE recurrence. The HR for VTE recurrence in female patients was 3.46. In the subgroup of unprovoked VTE recurrence in female patients, the HR was 5.94. To our knowledge, this is the first study in which *TLR9* rs5743836 polymorphism has been studied in VTE recurrence. Previously, there was a single study in which this polymorphism has been studied for its role in primary deep vein thrombosis (DVT) and no association was found between *TLR9* rs5743836 polymorphism and risk of DVT among a European population [27]. Recent studies have suggested that the risk factors for primary and recurrent VTE may differ [37, 38]. Furthermore, the above-mentioned study [27] included only male patients, while our results suggest that *TLR*9 rs5743836 polymorphism is only associated with risk of VTE recurrence in female patients but not in male patients. It is now well established that the risk of VTE recurrence varies according to the gender of the patients and risk factors for VTE recurrence are gender specific [32, 39]. Moreover, effects of sex hormones on inflammation in patients with and without *TLR*9 polymorphism may differ and thereby influence the risk of VTE recurrence in these patients [40, 41].

*TLR9* rs5743836 polymorphism has been shown to affect the transcription activity of *TLR*9, CC genotype (mutant) was associated with lower transcription activity as compared to TT genotype (wild-type) [25]. Gene knockout studies have shown a pivotal role of *TLR9* in VTE resolution [22, 23]. Taken together with our findings, this suggests that *TLR9* polymorphism may reduce the ability of VTE resolution and therefore may increase the risk of VTE recurrence.

We further analyzed *TLR*9 rs5743836 polymorphism in unprovoked VTE patients, which are known to have higher risk of VTE recurrence. We found a significant association between *TLR*9 rs5743836 polymorphism and VTE recurrence in female patients, independent of location of DVT, family history of VTE & mild and severe thrombophilia. These results show that *TLR*9 rs5743836 polymorphism may also be an independent risk factor for VTE recurrence in these higher risk female patients.

*TLR9* is one of the important members of pattern recognition receptors (PRRs) that are involved in thrombus resolution. Leukocytes express several pattern PRRs (including *TLR*9) that are activated by damaged associated molecular patterns (DAMPs) from cellular debris, causing the release of mediators that promote sterile inflammation [23]. Leukocytes, specially, neutrophils are known to be involved in the early thrombus resolution by promoting collagenolysis and fibrinolysis [42, 43] and monocytes are likely the most important cells involved in late VTE resolution [44]. Protective role of *TLR9*, as an integral part of thrombus resolution process, due to the rs5743836 polymorphism may be lost and therefore is associated with higher risk of VTE recurrence.

In conclusion, for the first time, we have analyzed *TLR9* rs5743836 polymorphism in recurrent VTE patients. Our results suggest that *TLR9* rs5743836 polymorphism is a potential marker for VTE recurrence in female but not in male patients. The findings shed new light on potential differential mechanisms by gender in the development of VTE recurrence as well as on future preventive strategies.

## Electronic supplementary material

Below is the link to the electronic supplementary material.

Supplementary material 1 (DOCX 13 KB)
