# Supplementary material for: Association between TLR9 rs5743836 polymorphism and risk of recurrent venous thromboembolism
Source: J Thromb Thrombolysis. 2017 Mar 20;44(1):130–8. doi: 10.1007/s11239-017-1491-3 (PMC5486891; doi:10.1007/s11239-017-1491-3)
Supplement: Supplementary file 1 — Supplementary material 1 (DOCX 13 KB) [file 11239_2017_1491_MOESM1_ESM.docx]

|  | **All patients** | | | | **Men** | | | | **Women** | | | |
| --- | --- | --- | --- | --- | --- | --- | --- | --- | --- | --- | --- | --- |
|  | **Multivariate** | **p*** | **Multivariate** | **p†** | **Multivariate** | **p*** | **Multivariate** | **p†** | **Multivariate** | **p*** | **Multivariate** | **p†** |
| Genotypes | HR (95% CI) |  | HR (95% CI) |  | HR (95% CI) |  | HR (95% CI) |  | HR (95% CI) |  | HR (95% CI) |  |
| **rs5743836** |  |  |  |  |  |  |  |  |  |  |  |  |
| TT | Reference |  | Reference |  | Reference |  | Reference |  | Reference |  | Reference |  |
| TC | 0.90 (0.59-1.36) | 0.611 | 0.88 (0.57-1.34) | 0.536 | 0.59 (0.32-1.11) | 0.102 | 0.60 (0.32-1.13) | 0.114 | 1.33 (0.76-2.33) | 0.315 | 1.27 (0.72-2.26) | 0.411 |
| CC | 1.08 (0.40-2.95) | 0.874 | 0.98 (0.36-2.68) | 0.967 | 0.33 (0.04-2.37) | 0.269 | 0.32 (0.04-2.36) | 0.267 | 3.60 (1.11-11.65) | **0.033** | 3.44 (1.05-11.26) | **0.042** |
| TT and TC | Reference |  | Reference |  | Reference |  | Reference |  | Reference |  | Reference |  |
| CC | 1.11 (0.41-3.01) | 0.835 | 1.01 (0.37-2.75) | 0.982 | 0.37 (0.05-2.65) | 0.322 | 0.37 (0.05-2.66) | 0.322 | 3.35 (1.05-10.74) | **0.042** | 3.23 (1.0-10.47) | **0.05** |

**Supplementary table 1:** Multivariate analyses of *TLR9* rs5743836 polymorphism in recurrent VTE patients with follow up from time of inclusion for this study and adjusted for duration of warfarin treatment.

p*= adjusted for duration of warfarin treatment, p†= Adjusted for duration of warfarin treatment, mild and severe thrombophilia, family history of VTE, location of VTE and acquired risk factors for VTE.
